# Role of Positive Age Beliefs in Recovery From Mild Cognitive Impairment Among Older Persons

**DOI:** 10.1001/jamanetworkopen.2023.7707

**Published:** 2023-04-12

**Authors:** Becca R. Levy, Martin D. Slade

**Affiliations:** 1 Department of Social and Behavioral Sciences, Yale School of Public Health, New Haven, Connecticut; 2 Department of Psychology, Yale University, New Haven, Connecticut; 3 Department of Internal Medicine, Yale School of Medicine, New Haven, Connecticut

## Abstract

This cohort study examines the contribution of positive age beliefs to recovery from mild cognitive impairment among older persons.

## Introduction

It is widely assumed that individuals who develop mild cognitive impairment (MCI) will not recover.^1^ Yet nearly half of older persons with MCI regain normal cognition.^2^ The reason for this improvement is not well understood. This study is the first, to our knowledge, to consider whether a culture-based factor—positive age beliefs—contributes to MCI recovery.

In previous experimental studies with older persons, positive age beliefs reduced stress caused by cognitive challenges, increased self-confidence about cognition, and improved cognitive performance.^3,4^ We therefore hypothesized that older persons with positive age beliefs would be more likely to recover from MCI and would do so sooner compared with individuals with negative age beliefs.

## Methods

The Yale University Human Investigation Committee approved this cohort study. All participants provided verbal informed consent. We followed the STROBE reporting guideline.

Participants were from the Health and Retirement Study, a national longitudinal survey. Inclusion criteria were age 65 years or older, baseline MCI as defined by Langa et al,^5^ at least 1 follow-up cognition assessment (measured with the Telephone Interview for Cognitive Status [TICS]) and a positive age-belief measure (assessed with the Attitude toward Aging subscale of the Philadelphia Geriatric Morale Scale [eg, disagreement with the item, “The older I get, the more useless I feel”]). Participants were dichotomized at the age-belief median into groups based on positive (<15) and negative (≥15) age beliefs. Covariates associated with MCI and/or age beliefs^1,2,3^ comprised baseline age, sex, self-reported race, education, marital status, smoking history, apolipoprotein E status, depression, cardiovascular and/or diabetes diagnosis, social isolation, sleep issues, and physical inactivity.

Cognitive recovery—the primary outcome—was defined as the first transition from MCI to normal cognition, using validated TICS cut points.^5^ Four nonoverlapping word lists were used to avoid practice effects. Seven data collection waves were performed (every 2 years, 2008-2020; <2% of person-survey waves were missing) (eAppendix in Supplement 1 provides additional details about data completeness and inclusion of all participants).

Analyses were conducted with 2-sided tests and SAS, version 9.4 (SAS Institute Inc). Data analyses were completed on February 15, 2023.

## Results

Our cohort study consisted of 1716 participants (953 women [55.5%] and 763 men [44.5%]), with a mean (SD) age of 77.8 (7.5) years (Table). Confirming our hypothesis, participants with MCI at baseline were significantly more likely to experience cognitive recovery if they had positive age beliefs at baseline (χ^2^ = 12.8; *P* < .001). A sensitivity analysis found that significant results did not change after adjustment for the number of participant TICS responses. The positive age-belief group had a 30.2% greater likelihood of recovery than the negative age-belief group; this recovery advantage persisted regardless of baseline MCI severity.

**Table.** Baseline Characteristics of Overall Sample and by Age-Belief Groups

Characteristic^a^	Participant group		
	Overall (N = 1716)	Positive age beliefs (n = 609)	Negative age beliefs (n = 1107)^b^
Age, y, mean (SD)	77.8 (7.5)	76.6 (7.1)	78.4 (7.7)^c^
Sex			
Women	953 (55.5)	335 (55.0)	618 (55.8)
Men	763 (44.5)	274 (45.0)	489 (44.2)
Race			
Black	369 (21.5)	148 (24.3)	221 (20.0)
White	1273 (74.2)	436 (71.6)	837 (75.6)
Other^d^	74 (4.3)	25 (4.1)	49 (4.4)
Hispanic ethnicity	193 (11.2)	68 (11.2)	125 (11.3)
Education			
Less than high school	687 (40.0)	223 (36.6)	464 (41.9)^c^
High school or greater	1029 (60.0)	386 (63.4)	643 (58.1)
Marital status			
Married	886 (51.6)	345(56.6)	541 (48.9)^c^
Not married	830 (48.4)	264 (43.4)	566 (51.1)
Smoking status			
Ever	1021 (59.8)	394 (57.4)	672 (61.2)
Never	686 (40.2)	259 (42.6)	427 (38.8)
Chronic disease^e^			
Yes	1389 (81.0)	471 (77.3)	918 (83.0)^c^
No	326 (19.0)	138 (22.7)	188 (17.0)
Depression^f^			
Yes	314 (18.5)	50 (8.3)	264 (24.0)^c^
No	1386 (81.5)	551 (91.7)	835 (76.0)
Apolipoprotein E			
ε4/ε4	19 (2.8)	10 (3.6)	9 (2.3)
ε3/ε4	180 (27.0)	84 (30.3)	96 (24.5)
ε3/ε3	363 (54.3)	143 (51.6)	220 (56.3)
ε2/ε4	20 (3.0)	10 (3.6)	10 (2.6)
ε2/ε3	83 (12.4)	29 (10.5)	54 (13.8)
ε2/ε2	3 (0.5)	1 (0.4)	2 (0.5)
Feel isolated			
Often	129 (7.8)	14 (2.4)	115 (10.9)^c^
Some of the time	459 (27.9)	89 (15.1)	370 (34.9)
Hardly ever or never	1059 (64.3)	485 (82.5)	574 (54.2)
Sleep issues, mean (SD)^g^	1.6 (0.6)	1.4 (0.6)	1.7 (0.6)
Physical activity^h^			
Daily	111 (11.2)	50 (13.0)	61 (10.1)
>1 times/wk	385 (38.9)	157 (40.9)	228 (37.6)
1 time/wk	157 (15.9)	53 (13.8)	104 (17.2)
1-3 times/mo	89 (9.0)	39 (10.2)	50 (8.2)
Never	248 (25.1)	85 (22.1)	163 (26.9)

^a^ Unless noted otherwise, data are presented as No. (%) of participants.

^b^ Participants were dichotomized at the age-belief median into groups based on positive (<15) and negative (≥15) age beliefs.

^c^ *P* < .05.

^d^ Includes American Indian or Alaska Native, Asian, and Native Hawaiian or other Pacific Islander.

^e^ Assessed with health care provider report giving a diagnosis of diabetes, heart disease, or hypertension.

^f^ Assessed with the 8-item Center for Epidemiological Studies Depression Scale cut-off score of 3 or greater for depression.

^g^ Assessed with the Jenkins Sleep Scale, which measures the frequency of sleep problems; mean scores range from 1 (rarely or never) to 3 (most of the time).

^h^ Assessed with a question about frequency of engaging in vigorous or moderate physical activity.

Also, as hypothesized, a proportional hazards model found that participants with positive age beliefs had a faster transition from MCI to normal cognition (hazard ratio, 1.26 [95% CI, 1.08-1.46]; *P* = .003). The positive age-belief group reached a 2-year recovery advantage over the negative age-belief group (Figure).

**Figure.**
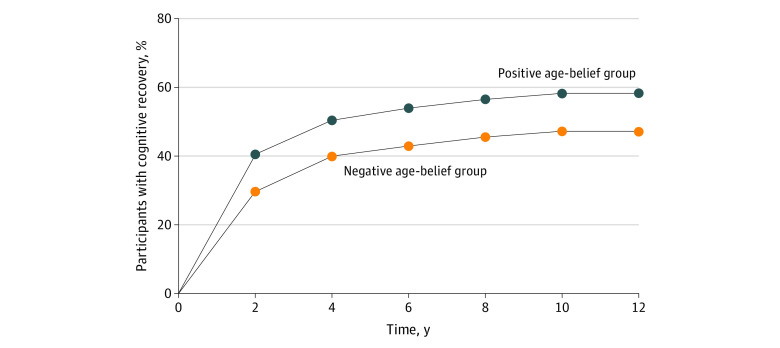
Role of Positive Age Beliefs in Recovery From Mild Cognitive Impairment to Normal Cognition Participants started at baseline with mild cognitive impairment. By tracking their cognition over 7 waves, it was found that those in the positive age-belief group had a 30.2% greater likelihood of cognitive recovery than those in the negative age-belief group.

Among participants with normal cognition or MCI at baseline, those with positive age beliefs had lower MCI prevalence (609 [16.3%] vs 1107 [24.0%]; χ^2^ = 76.4; *P* < .001) compared with those with negative age beliefs. Additionally, among participants with normal cognition at baseline and adjusting for all covariates, those with positive age beliefs were significantly less likely to develop MCI over the following 12 years compared with those with negative age beliefs (χ^2^ = 26.5; *P* < .001).

## Discussion

The findings of this cohort study suggest the importance of considering the role of culture, expressed here through age beliefs, in MCI development and reversal. A limitation is that we did not examine the mechanism of positive age beliefs in cognitive recovery. However, previous studies have reported that cognition is predicted by stress levels and health behaviors, both of which can be improved by positive age beliefs.^3,4,6^ Considering that positive age beliefs can be strengthened,^6^ our findings suggest that age-belief interventions at individual and societal levels could increase the number of people who experience cognitive recovery.

## Notes

Supplement 1. **eAppendix.** Data Analysis
eReference

Supplement 2.
Data Sharing Statement
