# Supplementary material for: Role of Positive Age Beliefs in Recovery From Mild Cognitive Impairment Among Older Persons
Source: JAMA Netw Open. 2023 Apr 12;6(4):e237707. doi: 10.1001/jamanetworkopen.2023.7707 (PMC10098975; doi:10.1001/jamanetworkopen.2023.7707)
Supplement: Supplement 1. — eAppendix. Data Analysis eReference [file jamanetwopen-e237707-s001.pdf]

Supplementary Online Content

Levy BR, Slade MD. Role of positive age beliefs in recovery from mild cognitive impairment among older persons. *JAMA Netw Open*. 2023;6(4):e237707. doi:10.1001/jamanetworkopen.2023.7707

eAppendix. Data Analysis

eReference

This supplementary material has been provided by the authors to give readers additional information about their work.

eAppendix. Data Analysis

Data Completeness and Inclusion of All Participants

In the primary analysis, all participants met the definition of mild cognitive impairment at baseline, and responded to the Telephone Interview for Cognitive Status (TICS) during one of the 7 waves of the study. They were followed for all waves available until they met the criterion for cognitive recovery or the last wave they gave data. In an analysis of the survey waves included for all participants, we found only 98 missing person-survey waves. These missing person-survey waves represented 1.95% of the possible person-survey waves in our study. Over 95% of participants had no missing data. Age-belief groups did not significantly differ in missing survey responses on either of these two measures of missing data.

Additionally, although there were few missing data points in our sample to prevent listwise deletion of participants with missing data, we utilized the missing-indicator method for categorical missing data.[1] This involved inserting a response level of "unknown" for these missing data points. As with imputation, it allows all other values of the participant to be utilized in the models.

eReference

1. Zhuchkova, S, Rotmistrov, A. How to choose an approach to handling missing categorical data: (un)expected findings from a simulated statistical experiment. Qual. Quant. 2022: 56:1-22.

© 2023 Levy BR et al. *JAMA Network Open*.
