# Supplementary material for: Role of Positive Age Beliefs in Recovery From Mild Cognitive Impairment Among Older Persons
Source: JAMA Netw Open. 2023 Apr 12;6(4):e237707. doi: 10.1001/jamanetworkopen.2023.7707 (PMC10098975; doi:10.1001/jamanetworkopen.2023.7707)
Supplement: Supplement 2. — Data Sharing Statement [file jamanetwopen-e237707-s002.pdf]

# Data Sharing Statement

Levy. Role of Positive Age Beliefs in Recovery From Mild Cognitive Impairment Among Older Adults. *JAMA Netw Open*. Published April 12, 2023. doi:10.1001/jamanetworkopen.2023.7707

**Data**
**Data available:** Yes
**Data types:** Deidentified participant data
**How to access data:** https://hrs.isr.umich.edu/data-products
**When available:** With publication

**Supporting Documents**
**Document types:** None

**Additional Information**
**Who can access the data:** Dataset will be available to everyone.
**Types of analyses:** It will be available for all purposes.
**Mechanisms of data availability:** Survey data, the data dictionary and supporting documentation are publicly available at the Health and Retirement Study website that is maintained by the University of Michigan. There are no restrictions on the use of the data.
